# Effect and Process Evaluation of a Smartphone App to Promote an Active Lifestyle in Lower Educated Working Young Adults: Cluster Randomized Controlled Trial

**DOI:** 10.2196/10003

**Published:** 2018-08-24

**Authors:** Dorien Simons, Ilse De Bourdeaudhuij, Peter Clarys, Katrien De Cocker, Corneel Vandelanotte, Benedicte Deforche

**Affiliations:** ^1^ Unit Health Promotion and Education Department of Public Health Ghent University Ghent Belgium; ^2^ Physical Activity, Nutrition and Health Research Unit Faculty of Physical Education and Physical Therapy Vrije Universiteit Brussel Brussels Belgium; ^3^ Department of Movement and Sport Sciences Faculty of Medicine and Health Sciences Ghent University Ghent Belgium; ^4^ Physically Active Lifestyles Research Group (USQ PALs) Institute for Resilient Regions University of Southern Queensland Springfield Central Australia; ^5^ Physical Activity Research Group School for Health, Medical and Applied Science Central Queensland University Rockhampton Australia

**Keywords:** mHealth, mobile apps, active transport, Fitbit, accelerometers, mobile phone, emerging adulthood, physical activity intervention, health promotion

## Abstract

**Background:**

Mobile technologies have great potential to promote an active lifestyle in lower educated working young adults, an underresearched target group at a high risk of low activity levels.

**Objective:**

The objective of our study was to examine the effect and process evaluation of the newly developed evidence- and theory-based smartphone app “Active Coach” on the objectively measured total daily physical activity; self-reported, context-specific physical activity; and self-reported psychosocial variables among lower educated working young adults.

**Methods:**

We recruited 130 lower educated working young adults in this 2-group cluster randomized controlled trial and assessed outcomes at baseline, posttest (baseline+9 weeks), and follow-up (posttest+3 months). Intervention participants (n=60) used the Active Coach app (for 9 weeks) combined with a Fitbit activity tracker. Personal goals, practical tips, and educational facts were provided to encourage physical activity. The control group received print-based generic physical activity information. Both groups wore accelerometers for objective measurement of physical activity, and individual interviews were conducted to assess the psychosocial variables and context-specific physical activity. Furthermore, intervention participants were asked process evaluation questions and generalized linear mixed models and descriptive statistics were applied.

**Results:**

No significant intervention effects were found for objectively measured physical activity, self-reported physical activity, and self-reported psychosocial variables (all *P*>.05). Intervention participants evaluated the Active Coach app and the combined use with the Fitbit wearable as self-explanatory (36/51, 70.6%), user friendly (40/51, 78.4%), and interesting (34/51, 66.7%). Throughout the intervention, we observed a decrease in the frequency of viewing graphical displays in the app (*P*<.001); reading the tips, facts, and goals (*P*<.05); and wearing the Fitbit wearable (*P*<.001). Few intervention participants found the tips and facts motivating (10/41, 24.4%), used them to be physically active (8/41, 19.6%), and thought they were tailored to their lifestyle (7/41, 17.1%).

**Conclusions:**

The lack of significant intervention effects might be due to low continuous user engagement. Advice or feedback that was not perceived as adequately tailored and the difficulty to compete with many popular commercial apps on young people’s smartphones may be responsible for a decrease in the engagement. A stand-alone app does not seem sufficient to promote an active lifestyle among lower educated working young adults; therefore, multicomponent interventions (using both technological and human support), as well as context-specific sensing to provide tailored advice, might be needed in this population.

**Trial Registration:**

ClinicalTrials.gov NCT02948803; https://clinicaltrials.gov/ct2/show/results/NCT02948803 (Archived by WebCite at http://www.webcitation.org/71OPFwaoA)

## Introduction

Insufficient physical activity has been estimated to cause 6%-10% of the major noncommunicable diseases such as coronary heart disease, type 2 diabetes, and breast and colon cancers and 9% of premature mortality [1]. Globally, up to 38% of young adults (aged 15-29 years) are physically inactive [2]. In Belgium, approximately 50% of 15- to 24-year-old individuals do not reach the recommended levels of physical activity [3], which is estimated to increase the all-cause mortality risk by 11.4% [1]. Young adulthood comprises many life changes (ie, changes in education, employment, and place of residence) [4,5], which have been shown to be associated with decreases in overall physical activity as well as in different types of physical activity, such as active transport [6-9]. Active transport represents an opportunity to include physical activity in the busy daily lives of young adults [2]. There is a need to improve overall physical activity during young adulthood as young adults’ behaviors are likely to track into adulthood [10,11]. Intervening during this life stage may facilitate positive behavior changes and improved health beyond young adulthood [12]. A large US cohort study has showed that a healthy lifestyle (ie, high physical activity levels and healthy weight) in young adulthood is strongly associated with a low risk of cardiovascular disease in middle-aged adults [13]. Young adults who do not complete higher education (college or university) and who start employment around the age of 18 years have an even higher risk for inactive lifestyle because of their lower educational attainment [14]. Among adults of all ages, lower levels of education have been associated with lower levels of overall physical activity [5,15], less active transport [16,17], higher levels of overweight or obesity, and the prevalence of common chronic diseases [18]. Specific research on young adults is scarce and mostly focused on students as they are easier to recruit through university and college settings [4]. Therefore, there is a need to promote an active lifestyle in the underresearched target group of lower educated working young adults.

Among all strategies to promote physical activity, the use of mHealth approaches is promising, especially among young adults. mHealth includes mobile technologies such as phones, tablets, and tracking devices that can be used to support and improve public health practice [19]. Mobile Smartphones are immensely popular worldwide and are most frequently used by young adults compared with other age groups [20-23]. In the United States [20] and Belgium [24], respectively, 85% and 80% of young adults own a smartphone. Moreover, in Belgium in 2015, 96% of 16-to-34-year-old individuals and 87% of adults with a low educational level used a mobile phone or smartphone [25]. Smartphone apps can measure health behaviors such as physical activity and provide feedback in real time; provide interactive, individualized, and automatically generated content; and deliver materials on a device (ie, smartphone) that is already carried by the individual [26]. In two systematic reviews and a meta-analysis, it was concluded that smartphones and health and fitness apps have great potential as a tool for assessing and promoting physical activity in all age groups [27-29]. However, a recent review and meta-analysis of randomized controlled trials (RCTs) using mHealth technologies to influence physical activity (also in all age groups) concluded that current mHealth interventions have only small effects on physical activity [30] as differences between mHealth intervention groups and comparators did not reach the statistical significance. However, most of these interventions were based on short message service text messages, while apps could enable more comprehensive, interactive, and responsive intervention delivery [30]. Nevertheless, there is still considerable scope to improve the efficacy of app-based interventions. In addition, process evaluations are necessary to identify factors that influence user engagement and retention and ultimately intervention efficacy [29]. Nevertheless, few mHealth interventions have conducted process evaluations [29,31,32].

Promoting an active lifestyle in the underresearched target group of lower educated working young adults is important, and mobile technologies have great potential to assist. Therefore, we developed a new evidence- and theory-based smartphone app called “Active Coach,” which aims to promote an active lifestyle to lower educated working young adults [33]. Therefore, in this study, we aimed to examine the effect and process evaluation of the Active Coach app on objectively measured total daily physical activity; self-reported, context-specific physical activity; and self-reported psychosocial variables among lower educated working young adults.

## Methods

### Study Design, Recruitment, and Sample

This cluster RCT included the baseline (T0), posttest (T1, 9 weeks after the baseline), and follow-up measurements (T2, 3 months after posttest) in 2 different study conditions (intervention and control). The intervention group received a smartphone-based intervention to promote an active lifestyle using a newly developed Android app called Active Coach in combination with a wearable activity tracker. Conversely, the control group received a printed brochure with generic information and tips about a physically active lifestyle. This study was approved by the Ethics Committee of the University Hospital of Ghent University (B670201525362). The trial registration number is NCT02948803 (Clinicaltrials.gov).

We identified, via an internet search, suitable workplaces in Flanders (northern, Dutch-speaking part of Belgium) based on the presence of lower educated (no university or college degree) employees aged 18-30 years. To recruit participants with various educational levels and various types of jobs, a range of workplace types (shops, retail stores, catering industry, social employment businesses, factories, etc) were contacted. Of the workplaces contacted by us in June and July 2016 via email and phone with information about the study, 51% (36/70) replied positively. After providing more details during a second contact, 14% (5/36) workplaces were excluded because of a lack of lower educated young employees and 6% (2/36) eventually disagreed to participate (eg, practical issues, no time). The required sample size was based on previous research, a statistical program (GPower [34]), and additional calculations to account for clustering. An effect size of 0.18 was determined based on a meta-analysis of internet-delivered interventions to increase physical activity levels [32]. In this meta-analysis, an overall mean effect size of 0.14 was found. However, after specifying the intervention based on the study design, participant characteristics, and intervention features, a mean effect size of 0.18 was determined [32]. Without accounting for clustering, the total sample size was calculated at 82 (80% power at a significance level of .05 with 2 groups [intervention group and control group] and 3 repeated measurements). This result was in line with a previously conducted RCT to test the effectiveness of a smartphone app to promote physical activity (step count) in primary care [35,36]. To account for clustering, an intraclass correlation coefficient (ICC) of .025 was assumed, based on previous worksite intervention studies with health-related outcomes [37-40]. Research states that sample size estimates need to be inflated by a factor 1 + (*n* − 1)*r* (where *n* is the cluster size and *r* is the ICC) to appropriately account for the clustering in the data [41]. As we did not know the number of clusters beforehand, we performed the calculation with 10, 20, 30, 40, and 50 clusters; this resulted in sample sizes of 100, 121, 141, 162, and 182, respectively. Therefore, we aimed to achieve a sample size of at least 120. Eventually, we included 130 participants (intervention group, 60; control group, 70) from 29 clusters (workplaces) in this study. Eligible employees were recruited through a contact person (eg, human resources manager), if available. At many smaller workplaces, as no contact person was available, the employees were directly contacted by the researchers. The recruitment process was conducted by DS, assisted by master students and research colleagues.

Eligible clusters (workplaces) needed to employ lower educated working young adults (aged 18-30 years). Allocation was based on clusters (workplaces), which were randomly assigned following block randomization (restricted randomization) to the intervention or the control group. Block sizes varied randomly (2, 4, or 6), and for each block of clusters, half (1, 2, or 3) would be allocated to each arm of the study (intervention or control group). Eligible participants needed to be employed, between 18 and 30 years of age, lower educated (no university or college degree), currently not meeting the physical activity guidelines of 150 minutes of moderate-to-vigorous physical activity (MVPA) a week [42], and not using an activity tracker or not participating in a sports program (via a website, an app, or a sports center). Furthermore, they needed to be in possession of an Android smartphone.

### Procedures

**Figure 1:**
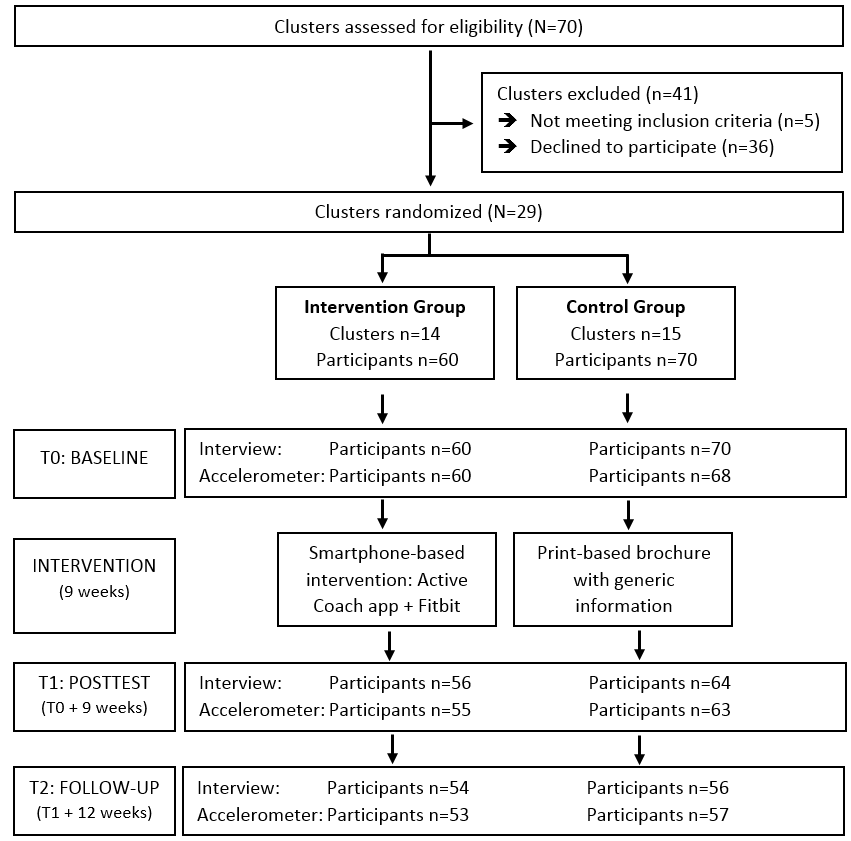
Flowchart of the smartphone-based intervention.

In September 2016, baseline measurements were performed (Figure 1). During the first visit to the workplaces, researchers and accompanying research assistants met every participant separately. The study details were explained to the participants based on an information letter. After agreeing to participate using a written informed consent, participants completed a brief questionnaire assessing sociodemographic data. Next, a face-to-face interview was conducted (mean duration: 30 minutes) to assess physical activity and psychosocial variables. In addition, participants were provided with an accelerometer (Actigraph GT3X+) for 1 week (7 days) and with explanations on how to wear it. One week later, the research team returned to the workplaces to collect the accelerometers. During that second visit, participants in the intervention group were asked to download the Active Coach app on their smartphone, and they received a wearable activity tracker (ie, Fitbit Charge). They were asked to use the Active Coach app and the wearable activity tracker for the next 9 weeks. However, participants in the control group only received a printed brochure with generic information and tips on a physically active lifestyle and did not use an activity tracker.

In November 2016, posttest measurements were performed. During this third visit to the workplaces (8 weeks after baseline), all participants were instructed again to wear an accelerometer. One week later (9 weeks after baseline, fourth visit to the workplace), the accelerometers were collected and a face-to-face interview (~30 minutes) was conducted to assess physical activity and psychosocial variables in both groups and process evaluation questions in the intervention group only. Notably, participants from the intervention group returned their Fitbit activity tracker.

In February 2017, follow-up measurements were performed. During this fifth visit to the workplaces (19 weeks after baseline, 11 weeks after posttest), all participants were instructed again to wear an accelerometer. One week later (12 weeks after posttest), the accelerometers were collected and a face-to-face interview (~30 minutes) was conducted to assess physical activity and psychosocial variables.

### Intervention

The development of the evidence- and theory-based app Active Coach has been described in detail elsewhere [33]. Briefly, a native Android app Active Coach was purposefully developed for lower educated working young adults using a stepwise approach, consisting of 4 steps, based on the Intervention Mapping Approach and the developmental steps for mHealth interventions [43,44].

In step 1, knowledge, attitude (perceived benefits and barriers), social support, and self-efficacy were selected as determinants important to promote an active lifestyle among lower educated working young adults. The selection was based on the existing literature, previous studies from our research group, the attitude-social influence-self-efficacy (ASE) model [45], and an exploratory qualitative study among the target group [33]. In step 2, self-regulation techniques (eg, goal setting and self-monitoring) were selected as evidence-based Behavior Change Techniques (BCTs) to convert the determinants into practical applications [46].

BCTs are the active component of an intervention designed to change behavior [47]. BCTs were selected on the basis of their previously demonstrated effectiveness and an exploratory qualitative study among the target group [33]. In step 3, the Active Coach app was developed. In step 4, the app was tested on errors; acceptability (is the target group willing to receive the strategies?); and feasibility (is it realistic to consider implementing the proposed strategies?) via (think aloud) interviews, a questionnaire, and Google Analytics. The app was accordingly adapted for the final version.

The app consists of a 9-week program with personal goals, practical tips, and scientific facts to encourage an active lifestyle. Users of the app receive tailored information about their goal, tips, and facts through notifications on their smartphone and messages in the Active Coach app. To ensure all-day and automatic self-monitoring of physical activity, the app works in combination with a wearable activity tracker, the Fitbit Charge. The Fitbit Charge is a wrist-worn activity tracker [48] that has been found to be valid and reliable for measuring step counts in healthy young adults [49]. The only measure tracked by the Fitbit Charge that was used in the Active Coach app was the number of steps.

During the registration in the Active Coach app, participants were provided with a choice on how to make their lifestyle more active: through overall physical activity or through active transport. Recreational physical activity was not added as a separate choice based on previous research and the results of the exploratory qualitative study during the development of the app stating that active transport can be integrated more easily into emerging adults’ busy lives compared with finding additional time to spend on recreational physical activity. After the registration, the app consists of a 9-week program. During those 9 weeks, participants’ physical activity (step count) was tracked by the Fitbit Charge and their active transport was tracked by inbuilt smartphone sensors (global positioning system and accelerometer). Regardless of the activity choice (overall physical activity or active transport), both behaviors were tracked automatically and were visible for the user in the app via graphical displays. However, other information and goals they received differed according to the chosen behavior. The first week of the 9-week program was a “monitoring week” during which the baseline activity level of users was assessed by the app. At the end of this week, a personal goal dependent on the baseline level of the chosen behavior (overall physical activity or active transport) was set by the app for the following week (eg, *Your goal for the next week is to try and walk 6000 steps each day*). Every day during the following 8 weeks, users received a notification on whether or not they had achieved their daily goal. Besides, their daily and weekly goal progression could also be viewed on the graphs in the app. In addition, users received feedback on their goal achievement at the end of each week. If they achieved their goal, they could increase it or maintain the same goal for the next week. If they did not achieve their goal, they could choose to decrease it or maintain the same goal for the next week. Additionally, users were asked why they did not achieve their goal to determine their perceived barriers. Different response options were shown for those who chose overall physical activity or active transport. This information was used to give users more personal feedback. Furthermore, every Monday and Friday during the 8 weeks after the baseline week, users received a notification with a practical tip, and every Wednesday, they received a notification with a scientific or educational fact to help and motivate them to reach their goal. The content of the tips and facts was tailored based on the information from the registration process (gender, type of job, overall physical activity or active transport, perceived benefits), goal achievement, and the selected barriers.

### Measures

#### Objective Measures

physical activity was assessed objectively using Actigraph GT3X+ accelerometers. Both reliability and validity of Actigraph accelerometers have been documented extensively [50-52]. Accelerometers were distributed in person, and participants were asked to wear the accelerometers on the right hip for 7 consecutive days during waking hours and remove it only for water activities (eg, swimming and showering). Uniaxial accelerometer data were collected in 15-second epochs and analyzed in 1-minute epochs. Nonwear time was defined as ≥60 minutes of consecutive zero counts. Only data of participants with at least 10 wearing hours for at least 3 days (as recommended to reliably predict physical activity behavior in young adults) were included in the analyses [53-55]. Furthermore, counts per minute were converted into minutes of light- (100-1951 counts/min), moderate- (1952-5724 counts/min), and vigorous-intensity physical activity (5725+ counts/min) according to the Freedson cut points for adults (based on uniaxial data) [50,56].

#### Self-Reported Measures

Sociodemographic variables were assessed using a paper-and-pencil questionnaire (only at baseline). Gender (male, female), age (open-ended), nationality (Belgian, other), workplace (open-ended), type of job (open-ended), employment duration (open-ended), and educational level (elementary, special secondary, vocational secondary, arts secondary, technical secondary, and general secondary education) were assessed. At all 3 time-points (T0, T1, and T2), participants reported their height (m) and weight (kg), which were used to calculate body mass index (BMI; weight/height²).

At all 3 time-points (T0, T1, and T2), a face-to-face interview was conducted. The interview consisted of the International Physical Activity Questionnaire (IPAQ) to assess context-specific physical activity. The Dutch IPAQ (long version, last 7 days interview version) has been validated in Flemish adults [57] and assesses the frequency (number of days in the last 7 days) and duration (hours and minutes per day) of physical activity in 4 different contexts (occupational physical activity, active transport, household physical activity, and recreational physical activity).

In addition, psychosocial variables were assessed during the interview. All questions on psychosocial variables were derived from previous studies [58-62]. The following psychosocial variables in relation to both physical activity and active transport were included because the Active Coach app focused on the following variables: social support, attitude (perceived benefits and perceived barriers), self-efficacy, and knowledge. A summary of the measures of psychosocial variables (ie, questions and scales) and Cronbach alpha for internal consistency is shown in Table 1. Averages of item scores were calculated. In addition, the intention to be physically active was also assessed. Participants could choose between “being physically active for more than 6 months or in the last 6 months” and “not being physically active but intend to start this month or but intend to start in the next 6 months or do not intend to begin.”

During the interview at baseline (T0), general smartphone usage was assessed by asking how many apps the participants (intervention and control group) had on their smartphone and how often they used these apps.

**Table 1:** Summary of psychosocial measures and Cronbach alpha values.

Scale (composition)	Question	Cronbach alpha		
		Pre	Post	Follow-up
Social support (1 item)^a^	How often do you have a physically active partner (someone to play sports with, to be physically active with, or to walk or cycle together with)?	N/A^b^	N/A	N/A
Perceived benefits (12 items)^c^	A benefit of being physically active (playing sports or walking or cycling somewhere) for me is (1) weight control; (2) less stress; (3) improved fitness; (4) improved health; (5) becoming more productive at work; (6) sleeping well; (7) social interaction; (8) fun; (9) low costs of active transport; (10) flexibility of active transport; (11) no traffic jams; (12) active transport is environment friendly	.78	.80	.84
Perceived barriers (13 items)^c^	Following reasons hinder me from being physically active (playing sports or walking or cycling somewhere): (1) no discipline; (2) no time; (3) no energy; (4) no company; (5) sweating; (6) no equipment; (7) no showers at work; (8) bad weather; (9) family demands; (10) too much work; (11) carrying luggage during active transport; (12) unsafe traffic; (13) no sports facilities	.76	.77	.75
Self-efficacy (8 items)^d^	How confident are you to be physically active (playing sports or walking or cycling somewhere) in the following situations: (1) bad weather; (2) busy for work; (3) darkness; (4) after a tiring day at work; (5) sweating; (6) friends or family demanding time; (7) stress; (8) no time	.78	.81	.82
Knowledge (1 item)	What do you think the recommended amount of moderate physical activity is? (1) 60 min on 1 d/wk; (2) 30 min on 3 d/wk; (3) 30 min on 7 d/wk; (4) 60 min on 7 d/wk; (5) I do not know	N/A	N/A	N/A

^a^5-point scale from 1 (never) to 5 (very often).

^b^N/A: Not applicable.

^c^5-point scale from 1 (strongly disagree) to 5 (strongly agree).

^d^5-point scale from 1 (know I cannot do it) to 5 (know I can do it).

Intervention group participants were asked process evaluation questions during the individual interview at posttest (T1). The use of the Fitbit Charge wearable (bracelet) was assessed by asking the participants whether (1) it was easy to use, (2) it was annoying to wear, (3) it was interesting to see the steps taken, (4) they would like to keep using it, and (5) they had technical issues (5-point scale from 1 [strongly disagree] to 5 [strongly agree]). In addition, the number of days (0-7) wearing the Fitbit wearable during the first 2 weeks, middle 4 weeks, and final 2 weeks of the intervention period was assessed, including the reasons why the participants did not wear it (open-ended question). Next, the use of the Active Coach app was assessed by asking the participants whether the app was (1) self-explanatory, (2) boring, (3) fun, (4) interesting, (5) complicated, (6) easy to use, and (7) motivating and (8) whether they encountered technical issues (5-point scale from 1 [strongly disagree] to 5 [strongly agree]). We also assessed which component of the app the participants liked best or least (open-ended question). Participants were asked how often (never, less than once a week, once a week, 2-4 times a week, every day, multiple times a day) and why or why not (open-ended question) they viewed the graphs during the first 3 weeks and the last 3 weeks of the intervention.

Similarly, participants were asked how often and why or why not they read the notifications on their smartphone and the app messages regarding tips, facts, and their goal. Furthermore, 4 statements about goal achievement and 7 statements about the tips and facts were assessed on a 5-point scale from 1 (strongly disagree) to 5 (strongly agree). Finally, participants were asked how often (5-point scale from 1 [never] to 5 [very often]) they used the official Fitbit app (which is needed to activate the wearable) and why they used it (open-ended question).

During the individual interview at the follow-up test (T2), participants (intervention and control groups) were asked whether they purchased or were planning on purchasing a consumer wearable activity tracker and why or why not (open-ended question).

#### Website Usage Statistics

Google Analytics [63] was used to obtain the app usage statistics and evaluate how participants used the Active Coach app. Google Analytics offers free tools to measure website and app data to gain usage insights. We evaluated the number of users, number and duration of app visits, and number and duration of screen views and events (ie, user interactions with content that can be tracked independently from a screen load, such as clicks on a notification or other element in the app).

### Analyses

Data were analyzed using IBM SPSS Statistics version 23 (Armonk, NY, United States). To check for differences between the control and intervention groups at baseline, independent-samples *t* tests and chi-square tests were conducted. We performed generalized linear mixed models analyses with a negative binomial distribution (log link) to assess the effectiveness of the intervention on the dependent variables of self-reported physical activity (minutes per week of occupational physical activity, active transport, household physical activity, recreational physical activity, and total physical activity [occupational physical activity + active transport + household physical activity + recreational physical activity]). The negative binomial distribution was used because the self-reported physical activity variables were positively skewed and contained a large number of zero values [64,65]. In addition, linear mixed models were performed to assess the effectiveness of the intervention on the following dependent variables: objective physical activity (minutes per day [not bouts] of light physical activity, moderate physical activity, vigorous physical activity, MVPA, total physical activity [light physical activity + moderate physical activity + vigorous physical activity], and steps per day) and self-reported psychosocial determinants (benefits, barriers, self-efficacy, intention, knowledge, and social support). In addition, the intervention group was split into participants who chose to focus on active transport and those who chose to focus on the overall physical activity in the Active Coach app to assess the effectiveness of the intervention in the corresponding types of self-reported physical activity (active transport among those who chose active transport and total physical activity among those who chose the overall physical activity). All models were controlled for gender (because of a significant baseline gender difference between the intervention and control groups) and included 3 hierarchically ordered levels: workplace, participant, and time. Intercepts were allowed to vary randomly at the workplace and participant level, and all slopes were assumed to be fixed. Generalized linear mixed models analyses allowed us to include all available measurements, even if participants completed only 1 or 2 measurements. Mixed models have advantages over fixed effects models in the treatment of missing values of the dependent variable. Mixed models are capable of handling the imbalance caused by missing observations and yield valid inferences if the missing observations are missing at random [66]. Furthermore, linear mixed models can handle correlated data such as responses of participants from the same workplace. To analyze the process evaluation measures, linear mixed models and descriptive statistics were calculated. *P* values<.05 were considered statistically significant.

## Results

### Study Sample

Table 2 shows the descriptive statistics of the total sample and group differences at the baseline. The total sample consisted of 130 participants with a mean age of 25 years, of which 48.5% (63/130) were males.

The mean BMI was 24.5 kg/m², with 25% (32/130) participants being overweight and 11% (14/130) being obese. Of all participants, 31.2% (39/130) had very low educational attainment (elementary and special secondary education), and the mean employment duration was almost 5 years. Of all participants, 45% (59/130) had a blue-collar job (eg, production line worker, warehouse manager, cleaner, and welder), 33% (43/130) had a pink-collar job (eg, sales, childcare worker, health care worker), and 21% (28/130) had a white-collar job (eg, administrative assistant). There was a gender difference between the 2 groups, with more males in the intervention group than in the control group. Participants in both groups had on average 10-20 apps on their smartphone and 85.4% (111/130) used these apps every day or multiple times a day. Dropout throughout the study period was rather limited (see Figure 1); it was mostly caused by changing jobs (participants were no longer available via the recruited workplace), being ill for a long period, and resistance toward wearing the Actigraph accelerometer. In the intervention group, 98% (59/60) participants had valid accelerometer data at baseline, 87% (48/60) at posttest, and 77% (41/60) at follow-up. In the control group, 93% (63/70) participants had valid data at baseline, 86% (54/70) at posttest, and 75% (43/70) at follow-up. The mean valid wear days were 5.7 (SD 2.3) days at baseline, 4.5 (SD 2.4) days at posttest, and 4.2 (SD 2.2) days at follow-up. The mean valid wear time was 13.5 (SD 1.4) hours/day at baseline, 13.1 (SD 1.5) hours/day at posttest, and 13.1 (SD 1.5) hours/day at follow-up.

### Effect Evaluation

Table 3 shows that there were no significant intervention effects for the objective physical activity data (light physical activity, moderate physical activity, vigorous physical activity, MVPA, total physical activity, and steps), for the self-reported physical activity data (occupational physical activity, active transport, household physical activity, recreational physical activity, and total physical activity), and for the self-reported psychosocial variables. However, significant time effects showed a decrease in self-reported total physical activity from baseline to follow-up and a decrease in the objective light intensity physical activity, total physical activity, and number of steps from baseline to posttest (the latter also showing a decrease in steps to follow-up).

**Table 2:** Descriptive statistics and group differences at baseline.

Characteristics	Total (n=130)	Intervention group (n=60)	Control group (n=70)	Group comparisons	*P* value
Males, n (%)	63 (48.5)	35 (58.3)	28 (40.0)	*χ*^2^_1_=4.4	.04
Age (years), mean (SD)	25.0 (3.0)	24.8 (3.1)	25.1 (3.0)	*t*_128_=−0.73	.47
Body mass index (kg/m²), mean (SD)	24.5 (4.5)	24.9 (4.5)	24.1 (4.4)	*t*_124_=0.94	.35
Elementary and special secondary education, n (%)	39 (31.2)	18 (30.5)	21 (31.8)	*χ*^2^_2_=0.03	.98
Employment duration (years), mean (SD)	4.8 (3.0)	4.8 (2.9)	4.8 (3.1)	*t*_127_=−0.05	.96

**Table 3:** Intervention and time effects of the objective physical activity, self-reported physical activity, and self-reported psychosocial variables.

Measures and groups			Baseline, mean (SE)	Posttest, mean (SE)	Follow-up, mean (SE)	*P* value	
						Time	Time × Group
**Objective**							
	**Light physical activity (min/d)**					.03^a^	.31
		Intervention group	320.8 (16.7)	291.7 (16.7)	296.7 (16.7)		
		Control group	338.9 (16.6)	329.7 (16.6)	333.4 (16.6)		
	**Moderate physical activity (min/d)**					.12	.56
		Intervention group	30.8 (3.4)	25.9 (3.4)	31.4 (3.4)		
		Control group	31.9 (3.8)	29.4 (3.8)	30.6 (3.8)		
	**Vigorous physical activity (min/d)**					.66	.26
		Intervention group	0.9 (0.5)	1.2 (0.5)	1.2 (0.5)		
		Control group	1.5 (0.5)	0.8 (0.6)	1.2 (0.6)		
	**Moderate-to-vigorous physical activity (min/d)**					.09	.66
		Intervention group	32.1 (3.6)	27.3 (3.6)	32.6 (3.6)		
		Control group	33.5 (4.0)	30.1 (3.9)	31.8 (3.9)		
	**Total physical activity (min/d)**					.01^a^	.36
		Intervention group	351.0 (18.2)	317.0 (18.2)	326.9 (18.2)		
		Control group	372.0 (18.4)	359.5 (18.4)	364.8 (18.4)		
	**Steps per day**					.003^a^	.64
		Intervention group	8619 (614)	7741 (614)	7767 (614)		
		Control group	8982 (644)	8061 (644)	8543 (644)		
**Self-reported**							
	**Occupational physical activity (min/wk)**					.28	.88
		Intervention group	530.8 (177.5)	484.4 (165.6)	377.5 (131.2)		
		Control group	619.0 (188.5)	517.2 (164.5)	322.0 (109.3)		
	**Active transport (min/wk)**					.91	.98
		Intervention group	68.4 (19.3)	74.2 (21.5)	73.7 (21.8)		
		Control group	81.3 (21.3)	86.9 (23.8)	96.0 (28.2)		
	**Household physical activity (min/wk)**					.10	.52
		Intervention group	109.9 (29.9)	116.5 (32.3)	92.9 (26.1)		
		Control group	162.3 (39.5)	158.6 (40.1)	85.7 (22.9)		
	**Recreational physical activity (min/wk)**					.59	.84
		Intervention group	189.5 (48.6)	139.2 (36.7)	130.1 (34.9)		
		Control group	179.7 (42.8)	158.9 (39.9)	165.3 (44.1)		
	**Total physical activity (min/wk)**					.02^a^	.81
		Intervention group	942.1 (153.6)	877.8 (145.1)	726.1 (121.5)		
		Control group	975.9 (141.8)	862.8 (130.8)	639.1 (101.5)		
	**Benefits^b^**					<.001^a^	.75
		Intervention group	3.7 (0.1)	3.4 (0.1)	3.4 (0.1)		
		Control group	3.7 (0.1)	3.5 (0.1)	3.4 (0.1)		
	**Barriers^b^**					.84	.82
		Intervention group	2.5 (0.1)	2.5 (0.1)	2.4 (0.1)		
		Control group	2.5 (0.1)	2.5 (0.1)	2.5 (0.1)		
	**Self-efficacy^c^**					.85	.41
		Intervention group	3.6 (0.1)	3.6 (0.1)	3.6 (0.1)		
		Control group	3.6 (0.1)	3.6 (0.1)	3.6 (0.1)		
	**Intention**					.55	.566
		Intervention group	3.3 (0.2)	3.1 (0.2)	3.1 (0.2)		
		Control group	3.4 (0.2)	3.3 (0.2)	3.3 (0.2)		
	**Knowledge (% correct answer)**					.002^a^	.514
		Intervention group	45.0%	51.7%	58.3%		
		Control group	42.9%	57.1%	50.0%		
	**Social Support^d^**					.94	.247
		Intervention group	2.6 (0.2)	2.5 (0.2)	2.7 (0.2)		
		Control group	2.8 (0.2)	2.9 (0.2)	2.7 (0.2)		

^a^*P*<.05 considered statistically significant.

^b^5-point scale from 1 (strongly disagree) to 5 (strongly agree).

^c^5-point scale from 1 (know I cannot do it) to 5 (know I can do it).

^d^5-point scale from 1 (never) to 5 (very often).

**Table 4:** Opinions about the use of the Fitbit Charge wearable and the Active Coach app in the intervention group.

Opinions		Strongly disagree, n (%)	Disagree, n (%)	Sometimes (dis)agree, n (%)	Agree, n (%)	Strongly agree, n (%)	Mean (SD)
**Fitbit wearable**							
	Easy	1 (1.8)	3 (5.5)	1 (1.8)	20 (36.4)	30 (54.5)	4.36 (0.91)
	Annoying	30 (54.5)	12 (21.8)	6 (10.9)	5 (9.1)	2 (3.6)	1.85 (1.16)
	Keep using	6 (10.9)	10 (18.2)	7 (12.7)	15 (27.3)	17 (30.9)	3.49 (1.39)
	Interesting	0 (0.0)	3 (5.5)	6 (10.9)	13 (23.6)	33 (60.0)	4.38 (0.89)
	Problems	27 (50.0)	8 (14.8)	6 (11.1)	7 (13.0)	6 (11.1)	2.20 (1.46)
**Active Coach app**							
	Self-explanatory	5 (9.8)	3 (5.9)	7 (13.7)	26 (51.0)	10 (19.6)	3.65 (1.16)
	Boring	20 (39.2)	15 (29.4)	8 (15.7)	6 (11.8)	2 (3.9)	2.12 (1.18)
	Fun	2 (3.9)	8 (15.7)	12 (23.5)	22 (43.1)	7 (13.7)	3.47 (1.05)
	Interesting	3 (5.9)	2 (3.9)	12 (23.5)	20 (39.2)	14 (27.5)	3.78 (1.08)
	Complicated	18 (36.0)	17 (34.0)	3 (6.0)	6 (12.0)	6 (12.0)	2.30 (1.39)
	Easy	1 (2.0)	4 (7.8)	6 (11.8)	22 (43.1)	18 (35.3)	4.02 (0.99)
	Motivating	7 (14.3)	12 (24.5)	4 (8.2)	16 (32.7)	10 (20.4)	3.20 (1.40)
	Problems	13 (26.5)	9 (18.4)	11 (22.4)	3 (6.1)	13 (26.5)	2.88 (1.55)

In addition, in both groups, perceived benefits significantly decreased from baseline to posttest and to follow-up, while knowledge regarding the physical activity recommendations significantly increased from baseline to posttest and to follow-up. After splitting the intervention group (data not shown) into participants who chose to focus on active transport and those who chose to focus on overall physical activity in the Active Coach app, no significant intervention effects were found in the corresponding types of self-reported physical activity (active transport among those who chose active transport and total physical activity among those who chose overall physical activity).

### Process Evaluation

At posttest (Table 4), 70%-90% of the intervention group indicated (agree + strongly agree) that the Fitbit wearable was easy to use (50/55) and not annoying to wear (42/55) and that it was interesting to look at the number of steps walked (46/55). In addition, more than half (32/55) of the intervention group participants indicated that they would like to keep using it. Technical problems when using the Fitbit wearable were encountered by 24% (13/55) of participants.

The frequency of wearing the Fitbit wearable decreased significantly (*P*<.001) from the first 2 weeks (mean 6.6 [SE 0.3] days/week) to the final 2 weeks (mean 4.6 [SE 0.3] days/week) of the intervention period. Participants indicated that they did not wear the Fitbit wearable because they forget to put it on (18/55, 32.7%) or forgot to recharge it (7/55, 12.7%), they found it annoying or unattractive to wear (5/55, 9.1%), they were not planning on being physically active (“what’s the point?”; 4/55, 7.3%), or they encountered technical problems (9/55, 16.4%).

Regarding the use of the Active Coach app (see Table 4), >70% of the intervention group participants found the app self-explanatory (36/51) and easy to use (40/51; agree + strongly agree). In addition, >60% of the participants found the app interesting (34/51), and approximately half of the intervention group found it fun (29/51) and motivating (26/51). The app was found boring by 15% (8/51) and complicated by 24% (12/51) of participants, and 32% (16/51) of participants had encountered technical issues when using the app. The high battery use (depending on smartphone type) due to Bluetooth connection with the Fitbit wearable was the most frequently mentioned technical issue.

Several participants indicated the graphs (graphical display of steps and minutes active transport) as the best feature of the app. The frequency of viewing the graphs decreased significantly (*P*<.001) from the first 3 weeks (mean 3.4 [SD 0.2] days/week) to the final 3 weeks (mean 2.7 [SD 0.2] days/week) of the intervention period. Participants indicated viewing the graphs less frequently toward the end of the intervention period because of decreased interest (n=12), they forgot about it (n=4), and the data were not up-to-date as they wore their Fitbit wearable less frequently (n=8).

During the intervention period, there was also a significant decrease in the frequency of reading the push notifications on the smartphone and messages (in the app) regarding participants’ goal, tips, and facts (Table 5). Participants indicated not reading the notifications or messages from the Active Coach app because they were getting lost among all other notifications (n=12; they did not see or read them, they swiped them [ie, moving a finger across the notification on the smartphone screen to quickly remove it]). Participants already received many notifications from others apps (eg, Facebook, Snapchat, and WhatsApp), and they did not want any extra notifications. In addition, they indicated that there was too much repetition among the notifications about their goal (n=5), and they mentioned a lack of interest in the notifications and messages (n=7).

Table 6 shows that >50% (25/45) of the intervention group participants tried to achieve their daily goal (agree + strongly agree) and >60% (30/45) found it motivating to have a goal. In addition, 40% (18/45) of participants found it helpful to receive daily feedback on their goal and 35% (15/45) found it helpful to receive weekly feedback on their weekly goal. The tips and facts were self-explanatory for 73% (30/41) of the intervention group participants, useful for 63% (26/41), and boring for 14% (6/41) of them. Half (20/41) of the intervention group participants thought the tips and facts were interesting, but only 24% (10/41) thought the tips and facts were motivating. Furthermore, the tips and facts were used to be physically active by only 20% (8/41) of the participants and only 17% (7/41) thought they were tailored and adapted to their life.

The official Fitbit app (this is the accompanying app with the Fitbit wearable) was sometimes used by 27.3% (15/55) and regularly used by 27.3% (15/55) of intervention group participants. The additional features within the Fitbit app that do not exist in the Active Coach app (eg, calories burned and sleep overview) were mentioned as reasons for using the Fitbit app. Furthermore, some participants found the Fitbit app more self-explanatory or opened the Fitbit app to enhance the syncing process with the Active Coach app.

At follow-up, 9 intervention group participants (3 in the control group) purchased a wearable activity tracker, and 14 intervention group participants (8 in the control group) were planning on purchasing it. Participants indicated that they found the wearable interesting, motivating, and useful when being physically active. Those who did not want to purchase it mentioned the high costs (n=13) and a lack of interest (n=12) as the reasons.

**Table 5:** Frequency of reading notifications and messages regarding goals, tips, and facts from the Active Coach app. 6-point scale: 1=never; 2=less than once a week; 3=once a week; 4=2-4 times a week; 5=every day; 6=multiple times a day.

Item	First 3 weeks, mean (SE)	Final 3 weeks, mean (SE)	*P* value (time)
Notification goal	2.95 (0.29)	2.57 (0.29)	<.001
Notification tips and facts	2.38 (0.22)	2.13 (0.22)	.004
Message goal	2.14 (0.21)	1.96 (0.21)	.005
Message tips and facts	2.39 (0.26)	1.99 (0.26)	.049

**Table 6:** Statements about goals, tips, and facts of the Active Coach app in the intervention group.

Statements	Strongly disagree, n (%)	Disagree, n (%)	Sometimes (dis)agree, n (%)	Agree, n (%)	Strongly agree, n (%)	Mean (SD)
I tried to achieve my daily goal.	3 (6.7)	8 (17.8)	9 (20.0)	15 (33.3)	10 (22.2)	3.47 (1.22)
I found it motivating to have a goal.	2 (4.4)	8 (17.8)	5 (11.1)	19 (42.2)	11 (24.4)	3.64 (1.17)
It was helpful that I received daily feedback about my daily goal.	8 (18.2)	9 (20.5)	8 (18.2)	12 (27.3)	6 (13.6)	2.91 (1.41)
It was helpful that I received weekly feedback about my weekly goal.	12 (27.9)	10 (23.3)	6 (14.0)	9 (20.9)	6 (14.0)	2.70 (1.44)
The tips and facts were interesting.	7 (17.1)	5 (12.2)	9 (22.0)	11 (26.8)	9 (22.0)	3.24 (1.39)
The tips and facts were clear, and I understood them.	7 (17.1)	1 (2.4)	3 (7.3)	16 (39.0)	14 (34.1)	3.71 (1.42)
I found the tips and facts motivating to be physically active.	10 (24.4)	12 (29.3)	9 (22.0)	7 (17.1)	3 (7.3)	2.54 (1.25)
The tips and facts were boring.	16 (39.0)	14 (34.1)	5 (12.2)	3 (7.3)	3 (7.3)	2.10 (1.22)
The tips and facts were tailored and adapted to my life.	12 (29.3)	12 (29.3)	10 (24.4)	5 (12.2)	2 (4.9)	2.34 (1.18)
The tips and facts were useful.	3 (7.3)	6 (14.6)	6 (14.6)	11 (26.8)	15 (36.6)	2.29 (1.31)
I used the info from the tips and facts to be physically active.	14 (34.1)	10 (24.4)	9 (22.0)	4 (9.8)	4 (9.8)	2.37 (1.32)

Results from Google Analytics showed that the Active Coach app did not crash a single time during the intervention period. In total, 59 people visited the app, with 59 people visiting the app in the first 3 weeks and 37 visitors in the last 3 weeks. The number of visits halved from 824 visits in the first 3 weeks to 403 visits in the last 3 weeks. The average duration of visiting the app was 1 minute 5 seconds (1 minute 19 seconds in the first 3 weeks vs 53 seconds in the last 3 weeks), and the average time users spent on a screen was 13 seconds (constant throughout the intervention period). Users viewed on average 5.3 screens per visit (repeated views of a single screen were counted). When examining events (user interactions which are not screen loads such as clicks on a notification or other element in the app), the event “daily goal not reached” occurred more often (242 times) compared with “daily goal reached or almost reached” (169 and 15 times, respectively). In addition, the event “weekly goal not reached” occurred more (39 times) compared with “weekly goal reached or almost reached” (35 and 26 times, respectively). The events of the tips (on Monday 77 times, on Friday 78 times) and facts (79 times) all occurred in similar amounts.

## Discussion

### Principal Findings

In this study, we investigated the effects and process evaluation of the Active Coach app, in combination with the Fitbit wearable activity tracker, in lower educated working young adults. The evidence- and theory-based Active Coach app was developed using a stepwise, user-centered approach to develop an app that is optimally suited to the needs and preferences of lower educated working young adults [33]. Nevertheless, results showed no significant intervention effects on the objective (light physical activity, moderate physical activity, vigorous physical activity, MVPA, total physical activity, and steps) and self-reported (occupational physical activity, active transport, household physical activity, recreational physical activity, and total physical activity) physical activity. Moreover, no significant intervention effects were found on the psychosocial variables.

Although no significant intervention results were found, intervention participants found the Active Coach app easy to use, self-explanatory, not complicated, and not boring. However, user engagement with the app showed significant decreases in the frequency of viewing the graphs and reading the messages and notifications throughout the intervention period. App engagement has previously been demonstrated to be positively associated with the intervention effectiveness and health behavior change [67-69]. Nevertheless, user engagement typically declines after the first few weeks in most eHealth and mHealth interventions [32,69-71]. This might be particularly true for lower educated working young adults as a qualitative study showed that young adults often lack commitment to using any particular app and they only tend to engage in transient and casual app use [22]. In addition, engagement with health interventions in general has typically been lower among those with lower levels of education [71].

Interactive app features such as notifications have been found to be essential for app engagement [72,73]; they are important as prompts for reuse of the app [74-76], and qualitative data also indicate that young adults want apps that include positively framed alerts or reminders (but not too frequently) [22,77]. In our study, participants mentioned that the notifications were getting lost among all the notifications from other apps on their smartphone. All young adults received many notifications from different apps such as communication apps (eg, WhatsApp) or social media apps (eg, Facebook and Snapchat), which were often perceived as more urgent and interesting compared with Active Coach app notifications. The app (particularly its notifications) was competing with many very popular and high-end commercial apps. Although young people have demonstrated high usage and adoption of app technology [22,29,78], this does not assure high engagement with health behavior apps. On the contrary, the abundance of popular commercial apps on smartphones might make it difficult for health behavior apps to cut through all the distractions and excitement created by other apps. All this makes young populations harder to reach using mHealth interventions.

Another explanation for the lack of intervention effects may be the fact that few intervention participants found the tips and facts motivating, used them to be physically active, and thought they were tailored and well suited to their life. Providing individually tailored feedback and advice (ie, based on users’ own characteristics [79]) has shown to be important for the engagement with and effectiveness of health behavioral change interventions [80-82]. However, this requires a knowledge of participants’ characteristics, which is typically gathered by manual data entry (eg, answers to a questionnaire). However, research has demonstrated that young adults want apps that require low effort, and this indicates the difficult balance between manual data entry burden and providing app users with personally tailored advice [22,73,83]. To limit the data entry burden, the personal advice in the Active Coach app was only tailored to a small extent [33]; unfortunately, this resulted in advice that was not perceived as motivational or useful. Therefore, advanced context sensing (using mobile or environmental sensors to automatically detect features of the person’s current behavior and circumstances) could be a solution for providing tailored advice with very low manual data entry burden in future studies, although the development of such apps is complex and costly [22,84-86].

A final explanation for the fact that the Active Coach app combined with a Fitbit wearable was not sufficient to encourage an active lifestyle among lower educated working young adults may be the lack of using additional intervention strategies in combination with the app and tracker. A recent review of mHealth interventions showed that multicomponent interventions yielded stronger intervention effects than stand-alone app interventions [29]. The use of multiple intervention strategies has been previously recommended to achieve long-term health behavior changes [87,88]. Integrating the Active Coach app into a multicomponent intervention in which digital and human support are combined might be necessary to increase the engagement in this particular target group. Especially, lower educated individuals may need an (expert) human coach who could reassure, guide, emotionally support them, or hold them accountable [69].

Notably, intervention participants were rather positive about the use of the Fitbit wearable. They found the wearable easy, user friendly, and interesting. In addition, many participants purchased or were planning on purchasing a wearable activity tracker at the end of the study. Previous research has also found positive evaluations of Fitbit use among both young and middle-aged adults [89-91]. Nevertheless, this did not influence physical activity levels; this might be because the frequency of wearing the Fitbit wearable during the intervention period decreased significantly. Participants mentioned not wearing it because, among other reasons, they forgot to put it on or forgot to recharge it. Although the use of the Fitbit wearable was included to ensure automated tracking of physical activity, which is important for user engagement with the app, it seemed that continuous engagement with the Fitbit wearable itself was also a problem. Similarly, previous studies on Fitbit use in young people found low engagement over time [92,93]. In addition, some participants did not see the need to wear the Fitbit wearable on days or moments when they were not planning on being physically active. They seemed to use the wearable to track planned sports activities, instead of monitoring everyday lifestyle physical activity at work, at home, or while traveling.

Lower educated working young adults are often employed within different occupational types, such as blue-collar work (ie, nonagricultural manual labor). Blue-collar jobs typically include more occupational physical activity compared with white-collar jobs. It could be suggested that lower educated working young adults do not benefit from an intervention to promote an active lifestyle because of their higher levels of occupational physical activity. However, occupational physical activity often includes activity patterns (heavy lifting, prolonged standing, repetitive work, and twisting or bending the back) that create opposing effects on health compared with other types of physical activity [94-96]. Moreover, high occupational physical activity has been associated with an increased risk of cardiovascular disease [97] and all-cause mortality, especially among employees with low physical fitness levels [95,96]. These contrasting health effects have been termed the physical activity health paradox [94,98], highlighting the importance of a good balance between physical fitness and physical work demands [96]. Encouraging an active lifestyle in lower educated working young adults could be beneficial for this balance.

We found decreases in both objective physical activity (light physical activity, total physical activity, and steps) and self-reported physical activity (total physical activity) in both the intervention and control groups; weather influences might have caused these physical activity declines. During baseline measurements (September 2016), the weather was unusually warm and dry for that time of year in Belgium (abnormally high mean temperature, 17.5°C, and sunshine duration and abnormally low total precipitation and wind speed), while it was normal during posttest (November 2016) and follow-up (February 2017) measurements and, therefore, markedly colder and wetter than that during baseline measurements [99]. Additional analyses (data not shown) support this hypothesis as we found a significant increase from baseline to posttest in the barrier related to bad weather and a significant decrease (all *P*<.05) in 4 benefits related to active transport. Weather has been shown to be a very important determinant for active transport [60,100,101] (see Table 1).

### Limitations and Strengths

A limitation of this study includes the relatively small sample size as multiple comparisons were not considered during sample size calculations. A larger sample size would have increased power and would have allowed for secondary outcome analyses such as gender differences in intervention effects. In addition, because the Active Coach app was specifically developed for Flemish lower educated working young adults, the generalizability of the evaluation results is limited. Furthermore, recall bias might have occurred as the IPAQ was conducted 3 times in 5 months. Participants might have become familiar with the questions and might have answered more accurately at posttest or follow-up (learning effect). In addition, participants may have been more physically active at baseline when wearing the accelerometer for the first time. It is possible that these 2 measurement effects contributed to the decreases in the objective physical activity and self-reported physical activity in both the intervention and control groups after the intervention.

Strengths of this study include the stepwise development of the Active Coach app, which was evidence and theory based and which was developed with frequent consultations with the target group. Nevertheless, we found no significant intervention effects. Due to the repetitive design of the pretesting study and the frequent contact with the researchers during the development, participants may have come to know what they had to do (use the app, read the notifications, give feedback, etc); they were more involved with the app and, therefore, provided a more positive reaction. The inclusion of both objective and self-reported measures to assess physical activityA is also a strength of this study. The elaborate process evaluation with both quantitative (eg, 5-point scales) and qualitative (open-ended questions) measures allowed for detailed insights into participants’ perceptions and experiences regarding using the Active Coach app [69]. This mHealth intervention study is unique as, to the best of our knowledge, it is the first to focus on the underresearched target group of lower educated working young adults.

### Conclusions

In this study, lower educated working young adults perceived the Active Coach app and its combined use with the Fitbit wearable as self-explanatory, easy, user friendly, and interesting. However, no significant intervention effects were found due to low continuous user engagement. The difficulty to compete with popular commercial apps on young people’s smartphones and the lack of highly tailored advice may have caused low engagement toward the end of the intervention. As a stand-alone app does not seem sufficient to promote an active lifestyle among lower educated working young adults, combining digital and human support in a multicomponent intervention and increased use of context sensing to provide tailored advice might be needed.

## Abbreviations

ASE: attitude-social influence-self-efficacy
BCT: Behavior Change Technique
BMI: body mass index
ICC: intraclass correlation coefficient
IPAQ: International Physical Activity Questionnaire
MVPA: moderate-to-vigorous physical activity
RCT: randomized controlled trial

## Appendix

Multimedia Appendix 1 CONSORT‐EHEALTH checklist (V.1.6.1).
